# Objectively measured physical activity and sedentary-time are associated with arterial stiffness in Brazilian young adults

**DOI:** 10.1016/j.atherosclerosis.2015.09.005

**Published:** 2015-11

**Authors:** Bernardo Lessa Horta, Beatriz D. Schaan, Renata Moraes Bielemann, Carolina Ávila Vianna, Denise Petrucci Gigante, Fernando C. Barros, Ulf Ekelund, Pedro Curi Hallal

**Affiliations:** a Post-Graduate Program in Epidemiology, Federal University of Pelotas, Brazil; b EndocrineDivision, Hospital de Clínicas de Porto Alegre, Porto Alegre, Brazil; c Department of Internal Medicine, Facultyof Medicine, Universidade Federal do Rio Grande do Sul, Porto Alegre, Brazil; d Department of Nutrition, Federal University of Pelotas, Brazil; e Medical Research Council, Epidemiology Unit, University of Cambridge, United Kingdom; f Department of Sport Medicine, Norwegian School of Sport Sciences, Oslo, Norway

**Keywords:** Vascular stiffness, Physical activity, Cohort studies

## Abstract

**Objective:**

To examine the associations between objectively measured physical activity and sedentary time with pulse wave velocity (PWV) in Brazilian young adults.

**Methods:**

Cross-sectional analysis with participants of the 1982 Pelotas (Brazil) Birth Cohort who were followed-up from birth to 30 years of age. Overall physical activity (PA) assessed as the average acceleration (m*g*), time spent in moderate-to-vigorous physical activity (MVPA – min/day) and sedentary time (min/day) were calculated from acceleration data. Carotid-femoral PWV (m/s) was assessed using a portable ultrasound. Systolic and diastolic blood pressure (SBP/DBP), waist circumference (WC) and body mass index (BMI) were analyzed as possible mediators. Multiple linear regression and g-computation formula were used in the analyses.

**Results:**

Complete data were available for 1241 individuals. PWV was significantly lower in the two highest quartiles of overall PA (0.26 m/s) compared with the lowest quartile. Participants in the highest quartile of sedentary time had 0.39 m/s higher PWV (95%CI: 0.20; 0.57) than those in the lowest quartile. Individuals achieving ≥30 min/day in MVPA had lower PWV (β = −0.35; 95%CI: −0.56; −0.14). Mutually adjusted analyses between MVPA and sedentary time and PWV changed the coefficients, although results from sedentary time remained more consistent. WC captured 44% of the association between MVPA and PWV. DBP explained 46% of the association between acceleration and PWV.

**Conclusions:**

Physical activity was inversely related to PWV in young adults, whereas sedentary time was positively associated.

Such associations were only partially mediated by WC and DBP.

## 1 Introduction

Physical inactivity is a well-established risk factor for non-communicable diseases and premature mortality [1]. It influences cardiovascular risk factors, such as blood pressure, lipid profile and adiposity, and consequently, increases the risk of coronary heart diseases [1], [2], [3]. In addition, new evidence suggests that the time spent in sedentary activities might be a risk factor for non-communicable diseases, independent of physical activity [4], [5].

Non-communicable diseases, particularly cardiovascular diseases are the main causes of death in high- [6] and middle-income countries [7]. The prevalence of cardiovascular risk factors such as diabetes, hypertension and obesity has increased worldwide, and these risk factors are associated with unfavorable changes in lifestyle behaviors such as an unhealthy diet and low levels of physical activity [6], [7]. Prevention of adult cardiovascular diseases implies detection and intervention in early life. Atherosclerosis is a chronic inflammatory disease that has a long asymptomatic phase [8] beginning in childhood and adolescence, and track into adulthood [9], [10].

Early detection of subclinical atherosclerosis and arteriosclerosis is possible through the evaluation of arterial stiffness, intima-media thickness and endothelial dysfunction, which can be measured by noninvasive, reproducible, and inexpensive techniques [11]. Arterial stiffness is associated with traditional cardiovascular risk factors, such as diabetes and hypertension [12], [13], and predicts increased risk of cardiovascular events and mortality [14]. Further, increased arterial stiffness in children and adolescents is associated with obesity and dyslipidemia [15], [16], [17].

Arterial stiffness is lower among individuals who regularly perform aerobic exercise [18], [19], and short-term aerobic exercise training reduces the stiffness in central arteries [19], [20], [21], however this effect cannot be maintained without continued exercise [22]. The association between exercise training and aortic stiffness observed in clinical studies are reinforced by observations in murine models suggesting that several genes identified involved in vasodilation and arterial elasticity are overexpressed by exercise [23].

Although an association between physical activity and arterial stiffness has been observed, few studies [24], [25] have measured physical activity using objective methods and it is unknown whether sedentary time is associated with arterial stiffness independent of moderate-to-vigorous physical activity (MVPA) and other potential confounding factors. We therefore examined the independent associations between objectively measured physical activity and sedentary time with pulse wave velocity (PWV) in Brazilian young adults who have been prospectively followed up since birth.

## 2 Methods

### 2.1 Subjects

In 1982, all hospital deliveries in Pelotas, a southern Brazilian city, were identified and those live borns (n = 5914) whose families lived in the urban area of the city were examined, and their mothers interviewed. These individuals have thereafter been followed on several occasions throughout their life-course (at the mean ages of 1, 2, 4, 13, 15, 18, 19 and 23 years). Further details about the methods of the cohort are available elsewhere [26], [27], [28]. The study was approved by the School of Medicine Ethics Committee of the Federal University of Pelotas. All participants signed the informed consent form.

Between June 2012 and February 2013, when participants were on average 30 years, we tracked the entire cohort using multiple strategies to locate cohort members. All participants were invited to visit the research clinic for interviews and a clinical examination.

### 2.2 Physical activity

Physical activity was measured using the GENEActiv accelerometer (ActivInsights Ltd., Kimbolton, UK). The monitor was worn on the non-dominant wrist. The GENEActiv activity monitor is waterproof and measures acceleration in three axes (x, y, z) with a sample frequency of 85.7 Hz. Data are stored directly as sampled from the MEMS chip and provided in units of *g* (1 *g* = 9.81 m/s^2^ = the magnitude of gravitational acceleration).

Individuals received the device during their visits to the research clinic. Participants who were disabled, living in others cities (except individuals who visited Pelotas weekly), with labor activity that did not allow the accelerometer use (i.e. baker, cook, mechanic, etc) were excluded from the measurements (825 exclusions, refusals and losses, including 72 pregnant women). Women who were pregnant during the clinical visit were not eligible and invited after delivery to wear the accelerometer. Physical activity was assessed between four and seven days, including at least one weekend day using a 24-h protocol. Participants who started their measurements on Mondays, Tuesdays or Wednesdays were monitored until the following Monday and, those who started their measurements on Thursdays, Fridays or Saturdays, were monitored until the following Wednesday. The first 10 h were excluded because this was the maximum period observed between initialization and attachment of the monitors.

The accelerometers were set up and downloaded in the GENEActiv software. The accelerometer data in binary format were analyzed with R-package GGIR (http://cran.r-project.org). The average magnitude of wrist acceleration over the measurement period normalized to a 24-h period after exclusion of invalid data segments was the main measure used. The signal processing scheme as carried out by GGIR included the following steps: verification of sensor calibration error using local gravity as a reference [29], detection of sustained abnormally high values, non-wear detection, calculation of the vector magnitude of body acceleration using the Euclidian Norm minus one (ENMO: $\sqrt{x^{2}+y^{2}+z^{2}}-1g$) with resulting negative values rounded up to zero, and imputation of invalid data segments by the average of similar time points on different days of the measurement.

Files were considered as valid if data were present for every 15-min period in a 24-h cycle (even when scattered over multiple days) and with calibration error lower than 0.02 *g* (after calibration error correction). Results are presented in milli-*g* (1 m*g* = 0.001 *g*) for readability reasons. A time window (60-min with 15-min moving increments) was classified as non-wear time if, for at least two out of the three accelerometer's axes, the standard deviation was less than 13 mg and the value range was less than 50 mg.

The summary measure ENMO was used as an indicator of average magnitude of dynamic wrist acceleration over the measurement period. Time spent in moderate-to-vigorous physical activity (MVPA) per day was estimated using an intensity threshold of 100 m*g* based on 5-s epoch data and 10 min bout durations in the minimum, and <20% of the data points below this threshold. Sedentary time was defined as the time spent below an intensity threshold of 50 mg, excluding the hours between 11:00 p.m. and 7:00 a.m. – assumed as sleeping period, measured in minutes/day [30]. The percentage of individuals who achieved the recommendation of at least 30 min/day spent in MVPA was calculated.

### 2.3 Pulse wave velocity

The carotid-femoral PWV (meters/second) was examined twice during the clinical visit using a portable ultrasound, Sphygmocor^®^ (Atcor Medical version 9.0, Sydney, Australia) in the supine position and measurements were taken in the right side. An electrocardiogram was registered at the same time. Duration of the examination was 10–15 min for each participant. The distance of pulse wave transit was measured by a flexible tape as the distance from suprasternal notch to femoral point of application of the tonometer and the distance from carotid point of tonometer application and the suprasternal notch. PWV was calculated by the software as the distance between the measurement sites divided by transit time delay between femoral and carotid pulse wave. The mean of measurements was used in the data analysis.

Training for PWV assessment was carried out in two days using volunteers. PWV was calculated by the software as the distance between the measurement sites divided by transit time delay between femoral and carotid pulse wave. The software evaluated the quality of each measurement according to the format of wave and synchronism with wave from electrocardiogram. This protocol followed recommendations from expert consensus document on arterial stiffness and the Research Applications Manual of SphygmoCor [31], [32].

### 2.4 Covariates and mediation analysis

Other characteristics evaluated in this study were: sex, skin color (self-reported), family income at birth, socioeconomic status (using the National Economic Indicator score at 30 years; obtained through factor analysis and based on the ownership of household goods) [33] and current smoking (self-reported). Information on skin color (assessed by the interviewer in the perinatal survey as white or nonwhite) for 115 participants was missing, and maternal skin color was used as a proxy. Due to this reason skin color was grouped in white and nonwhite.

Possible mediators evaluated in this study were: body mass index (BMI), waist circumference (WC), systolic (SBP) and diastolic blood pressure (DBP). BMI was calculated by dividing the weight in kg by the square of height in meters. Standing height was measured to the nearest 1 mm with barefooted subjects using a wooden stadiometer. Weight was assessed using the BodPod^®^ scale with a precision of 0.01 kg. Waist circumference was measured using a flexible tape (Cescorf^®^, Porto Alegre, Brazil) with an accuracy of 0.1 cm at the narrowest part of the trunk, identified as the midpoint between the lowest rib margin and the iliac crest. The measurement was taken after a gentle expiration twice. If the difference between the two measures was greater than 1 cm, two additional measures were taken. Blood pressure was measured twice, at the beginning and at the end of the anthropometric measurements, in the sitting position using a digital sphygmomanometer Omron model HEM-705CPINT (Omron, Beijing, China) on the left arm. The means of the two readings were used in the present analysis.

Daily energy intake (kcal) was estimated using a semi-quantitative food frequency questionnaire (FFQ) in a self-administered and digital version with 88 food items especially created for this cohort. Frequency of food consumption was asked in <once/month; 1–3 times/month; once/week; 2–4 times/week; 5–6 times/week; once/day; 2–4 times/day and ≥5 times/day. After, using photos of standard servings based on previous 24-h dietary recall (50 percentile), individuals answered if they usually ate a serving equal, greater or lower than shown in the picture. Because FFQs usually overestimate the food consumption, the lowest frequency chosen was considered for each food. Frequency was multiplied by 0.5, 1 or 1.5 if serving chosen was lower, equal or greater than the standard serving, respectively. Daily food frequency was obtained dividing the information in years by 365.24 days/year. Based on references available to estimate the macronutrients [34], [35], [36], the amount of carbohydrates, proteins and fats of each food item was obtained and it was possible to determine the daily energy intake by multiplying the amount of carbohydrates or proteins by 4 kcal and the amount of fats by 9 kcal. Individuals above 3 standard deviations from average of daily energy intake by sex were excluded as well as individuals above 2 standard deviations from average with BMI and physical activity inconsistent to the estimated food consumption - high energy intake/normal weight/low physical activity or low consumption/obesity/high physical activity – e.g. consumption of 6.000 kcal/day, normal weight and 0 min of moderate-to-vigorous physical activity per week (111 exclusions in 3646 individuals with information in FFQ).

### 2.5 Statistical analysis

**Fig. 1:**
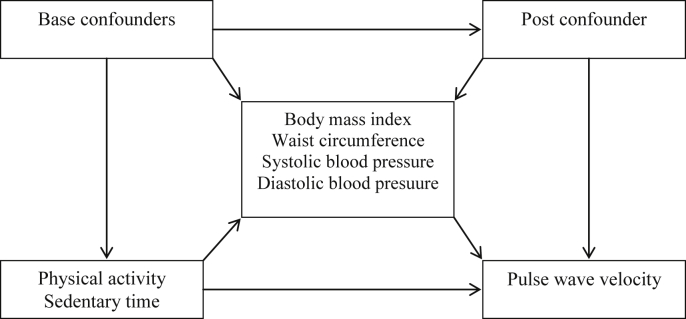
Direct acyclic graph of the effect of physical activity and sedentary time on pulse wave velocity (PWV).

**Fig. 2:**
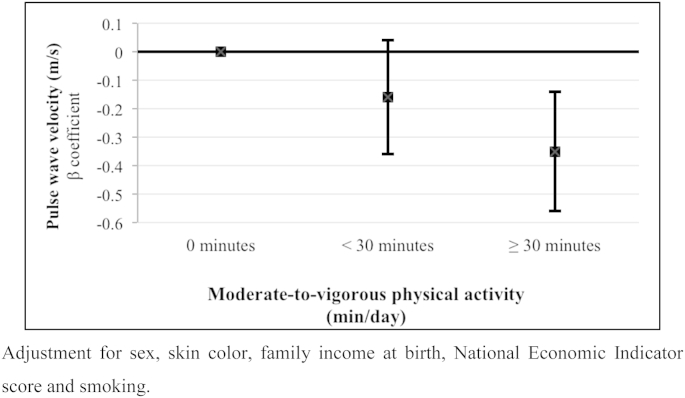
Pulse wave velocity (PWV, m/s) according to time spent in moderate-to-vigorous physical activity in young adults from the 1982 Pelotas Birth Cohort.

Data analysis was carried out using Stata 12.0 (StataCorp, College Station, TX, USA). Description of the sample with complete data of PWV was described in proportion or mean and standard deviation, according to type of variable. Linear regression models were used in the crude and adjusted analyses using all exposures in quartiles to assess possible dose-response. Statistical significance was obtained using two-sided Wald's tests. Possible confounders were: sex, skin color, family income at birth, socioeconomic status and current smoking. The association of MVPA and sedentary time with PWV was also mutually adjusted between them. We evaluated whether the associations were modified by sex. Mediation analyses for BMI, WC, SBP and DBP were carried out using the g-computation formula [37]. This analysis estimates the direct effect of acceleration, sedentary time and MVPA on PWV at 30 years, and the indirect effect that was mediated through BMI, WC, SBP and DBP, separately. Sex, skin color, family income at birth, socioeconomic status and current smoking were considered as base confounders, whereas daily energy intake was considered as post confounder. Fig. 1 shows the direct acyclic graph of this analysis. G-computation formula adjusts the estimates for base confounders – variables that affect both main exposures and outcome – and post confounders – variables not previously included in the model that can be affected by exposures and related to the mediating variable. In this analysis, sex, skin color, family income at birth, socioeconomic status and current smoking were considered as base confounders. Daily energy intake was considered as post confounder. Total effect (relationship between each exposure and outcome, with and without influence of the mediator), direct effect (relationship between each exposure and outcome without influence of the mediator), indirect effect (relationship between each exposure and outcome with influence of the mediator – captured by the mediator). A p-value of 0.05 was used to assign statistical significance (Fig. 2).

## 3 Results

At 30 years of age, 3701 individuals were located and examined representing a follow-up rate of 68.1% (following exclusion of 325 known deaths). Due to practical reasons (delay in the arrival of the equipment and tonometer with problem later) PWV was measured in 1576 participants (42.6% of those interviewed) and data on physical activity were available in 2740 participants. In total, 1241 participants provided information on both physical activity and PWV and were included in the present analyses. Table 1 shows that the proportions of females among those who were examined at 30 years and those with complete data were 52% and 49%, respectively (p = 0.02). Individuals with and without complete information from accelerometry and PWV were also statistically different concerning skin color, family income at birth, smoke, sedentary time and waist circumference. Spearman correlation coefficient between MVPA and sedentary time was −0.45 (p < 0.001)

Because there was no evidence of interaction with sex in the association between accelerometry and PWV (p-values for interaction ranging from 0.18 to 0.58), all analyzes were performed sex combined adjusting for sex as a confounder. Fig. 1 shows that PWV among those individuals who spent less than 30 min/day in MVPA was not statistically lower than that observed among those with 0 min/day in MVPA. However, PWV was lower among individuals who achieved the recommendation of ≥30 min/day of MVPA [−0.35 m/s (95%CI: −0.56; −0.14)].

Acceleration was inversely related to PWV - subjects in the highest quartile of acceleration had on average −0.37 m/s [β = −0.37 m/s (95%CI: −0.56; −0.19)] of PWV than those in the first quartile. Individuals in the third and fourth quartile of time spent in MVPA had similar reductions in PWV. Conversely, participants in the highest quartile of sedentary time had on average 0.36 m/s higher PWV [β = 0.36 m/s (95%CI: 0.17; 0.52)] than those in the lowest quartile. Association between MVPA in quartiles and PWV remained after adjustment for sedentary time (p = 0.046), although beta coefficients reduced for −0.18 m/s in the two highest quartiles [β = −0.18 m/s (95%CI: −0.36; −0.01–95%CI: −0.37; 0.01, in third and fourth quartile, respectively)], and 95% confidence interval was not statistically significant in the fourth quartile. On the other hand, PWV was substantially higher in the highest quartile of sedentary time, even after adjustment for MVPA [β = 0.28 m/s (95%CI: 0.09; 0.47)] (Table 2).

Table 3 shows that DBP captured about 46% of the effect of acceleration on PWV. Concerning the association between MVPA and PWV, 44% of this association was explained by WC, whereas DBP captured only a small proportion (27%) of the association between sedentary time and PWV.

## 4 Discussion

In an analysis involving 1259 young adults from Brazil, we observed that subjects who were more physically active had lower PWV. Subjects who spent more than 30 min/day in MVPA had a lower PWV. In addition, PWV was positively associated with sedentary time. Coefficients from association between MVPA and sedentary time and PWV changed in mutually adjusted analyses and were more consistent for results using sedentary time. WC and DBP were important mediators in the association of MVPA and sedentary time with PWV.

Similarly to our study, an inverse association between physical activity and the central augmentation index has been observed among adults aged 55 years, on average [24]. However, one study carried out among children failed to observe an association between physical activity intensity and stiffness in the carotid arteries [38]. These null findings are possibly related to the small sample studied and the low probability that these children had to develop any arterial derangement at this age [38]. Vascular changes associated with the aging process are progressive, involve hypertrophy and hyperplasia of smooth muscle cells within the vascular tree, coupled with the modification of matrix proteins, and may occur before hypertension begins [39]. The continuous deposition of a variety of proteins, including collagen, coupled with progressive loss of the elastic matrix will result in arterial stiffening [40]. In normotensive volunteers, blood pressure increases slowly from young to older ages, resulting in age-associated increases of arterial stiffness [41]. These information support the idea that, although vascular changes begin early in life – what was seen in this study, it is expected that they could be incipient at that age. Our study draws attention to the influence of an active behavior on vascular stiffness in the first decades of life.

Recently, sedentary behavior has been recognized as a risk factor for obesity, diabetes and cardiovascular diseases, independent of MVPA [42]. Sedentary behavior is a different construct than physical inactivity, with both behaviors having different determinants [43]. Interestingly, in the present study both behaviors were shown to be associated with PWV: sedentary time was positively related to PWV and MVPA was inversely associated with PWV. Given the trend towards increased sedentary behaviors in children and adolescents [44], our findings among young adults suggest that more focus should be directed at increasing the volume of MVPA alongside decreasing sedentary behavior. A minimum of 30 min/day in MVPA resulted in improvements in arterial distensibility of our population.

There is debate in the literature whether the health effects of sedentary time are really independent of those of physical activity [45]. In order to deal with this issue, we present results adjusting the two variables for each other, as well as the correlation between them. Current results showed a negative correlation (r = −0.45) between MVPA and sedentary time. The association between both measurements and PWV remained statistically significant after adjustment one for each other, though beta coefficients were slightly higher for sedentary time.

Training positively affect aortic PWV [46], [47] and central aortic distensibility [19]. This effect is due to qualitative structural elements (interstitial collagen of the arterial wall that can react producing advanced glycation end-products) and functional elements (vasoconstrictor tone, endothelial function) [47] that are influenced by physical activity. Aortic PWV modulation by exercise was shown to occur in parallel to changes in plasma concentrations of endothelin-1 (ET-1), independent of blood pressure, suggesting that ET-1 may be involved in the adaptations of arterial stiffness to exercise training [46]. Moreover, exercise can reduce chronic inflammation, improving endothelial function [48]. In addition, sedentary behavior may reduce glucose transporter protein content [49], leading to insulin resistance [50], which is associated with arterial stiffness independent of glucose tolerance status [51].

Waist circumference captured part of the relationship between MVPA and PWV. This can be due to the effect of physical activity on weight loss and in visceral fat [52], which in turn, are positively related to arterial stiffness [53], [54].

High levels of sedentary time can coexist with high levels of MVPA [55]. In this case, it may be that sedentary time substitutes light-intensity physical activity [56] that, in turn, has been beneficially associated with health outcomes, such as blood glucose [57]. There is some evidence that not only the decrease in the total amount of physical activity, including light-intensity activity, is a concern for health, but also the sedentary time itself, operating through other mechanisms, such as decreasing lipoprotein lipase, which maintains cardiometabolic homeostasis and regulate lipid concentrations [58].

Possible limitations of our study include losses to follow up, particularly because due to operational reasons, we were unable to measure PWV and physical activity from accelerometry in all those followed up at 30 years. However, since this is an association study, these losses do not affect the relevance of our findings. In addition, though with statistical significance, the differences found showed very similar distribution of the individuals in the variables investigated. Moreover, the amount of days/time measure was variable depending on the day of the week the person was included. In addition, some had more weekdays, others less. Since the amount of activity varies between weekdays and weekend, this could have influenced the results. Another issue to be considered is that since only a couple of days/hours were collected, this may not represent routine life. Finally, since the changes in PWV are a chronic process, and the amount of activity may change over the years, it may well happen that the PWV measured today reflects the history of physical activity over the last years, which may not be related to today's activity.

The most important strengths of the current study are the large and representative sample of young adults who had their levels of MVPA and sedentary time objectively evaluated by the use of accelerometer. In addition, early vascular disease was objectively assessed. Moreover, the availability of information to perform a mediating analysis helped to elucidate the pathways that connect physical activity and sedentary time to PWV.

In conclusion, the findings showed an inverse association between objectively measured physical activity and PWV, as well as a positive association between sedentary time and PWV. Such associations were only partially mediated by WC and DBP. Reducing sitting time and promoting physical activity is essential to help prevent the incidence of cardiovascular diseases across the lifespan.

## Conflict of interest

None declared.

## Figures and Tables

**Table 1:** Comparison between all participants of the 30 years follow up visit and those with measurements of pulse wave velocity (PWV). The 1982 Pelotas (Brazil) Birth Cohort.

	Cohort members followed-up in 2012/3		Participants with complete information		p
n	%	n	%
**Gender**					0.02
Males	1787	48.3	633	51.0	
Females	1914	51.7	608	49.0	
**Skin color**					0.04
White	2817	76.1	919	74.0	
Non-white	884	23.9	322	26.0	
**Family income at birth** (minimal wages)					0.02
<1	730	19.8	252	20.4	
1.1–3	1816	49.3	642	51.9	
3.1–6	721	19.6	223	18.0	
6.1–10	222	6.0	70	5.7	
>10	195	5.3	49	4.0	
**Current smoker**	854	23.5	332	26.8	0.001
**Overweight/Obese**	2042	56.6	715	57.7	0.4
**Sedentary time** (min/day)					0.02
1st quartile (317.8–623.9)	685	25.0	342	27.5	
2nd (624.0–684.8)	687	25.1	301	24.3	
3rd (684.8–739.7)	688	25.1	310	25.0	
4th quartile (739.8–952.1)	678	24.8	287	23.2	
**MVPA** (min/day)					0.08
1st quartile (0–5.3)	685	25.0	300	24.3	
2nd (5.3–16.3)	683	25.0	287	23.2	
3rd (16.3–34.6)	681	25.0	321	26.0	
4th quartile (34.6–379.6)	681	25.0	329	26.6	
**Waist circumference** (cm) [n - mean(sd)]	3567	84.8 (12.6)	1240	85.4 (12.6)	0.04
**Systolic blood pressure** (mmHg) [n - mean(sd)]	3592	121.2 (13.8)	1234	121.5 (13.5)	0.4
**Diastolic blood pressure** (mmHg) [n - mean(sd)]	3592	75.4 (9.3)	1234	75.2 (8.9)	0.4
**Daily energy intake** (kcal) [n – mean(sd)]	3535	2333.2 (1233.8)	1192	2365.0 (1263.0)	0.3

MVPA – Moderate-to-vigorous physical activity.

**Table 2:** Cross-sectional associations between (a) objectively measured physical activity; (b) sedentary time and pulse wave velocity (PWV) in young adults belonging to the 1982 Pelotas (Brazil) Birth Cohort.

	N	Crude		Model 1		Model 2	
β (95%CI)	p	β (95%CI)	p	β (95%CI)	p
**Acc** (m*g*)	1241		0.001		<0.001		
1stquartile		–		–		–	
2nd		−0.03 (−0.20; 0.15)		−0.06 (−0.24; 0.13)		–	
3rd		−0.21 (−0.39; −0.04)		−0.24 (−0.42; −0.06)		–	
4th quartile		−0.30 (−0.47; −0.12)		−0.37 (−0.56; −0.19)		–	
**MVPA** (minutes)	1238		0.004		0.001		0.05
1st quartile		–		–		–	
2nd		0.00 (−0.19; 0.17)		−0.02 (−0.20; 0.15)		0.02 (−0.16; 0.20)	
3rd		−0.22 (−0.39; −0.05)		−0.25 (−0.42; −0.07)		−0.18 (−0.36; −0.01)	
4th quartile		−0.24 (−0.41; −0.07)		−0.29 (−0.46; −0.11)		−0.18 (−0.37; 0.01)	
**Sedentary time** (minutes)	1259		0.002		0.001		0.03
1st quartile		–		–		–	
2nd		0.07 (−0.10; 0.23)		0.09 (−0.08; 0.26)		0.06 (−0.12; 0.23)	
3rd		0.14 (−0.02; 0.31)		0.16 (−0.01; 0.33)		0.12 (−0.06; 0.29)	
4th quartile		0.32 (0.15; 0.49)		0.35 (0.17; 0.52)		0.28 (0.09; 0.47)	

Acc – Acceleration by raw data accelerometry; MVPA – Moderate-to-vigorous physical activity.

Model 1: Adjustment for sex, skin color, family income at birth, National Economic Indicator score and smoking.

Model 2: Adjustment for Model 1 + MVPA/sedentary time.

P-values tested the heterogeneity between groups.

**Table 3:** Total, direct and indirect effects of objectively measured physical activity and sedentary time on pulse wave velocity of young adults considering body mass index, waist circumference and blood pressure as possible mediators.

	Pulse wave velocity (PWV, m/s)		
Total effect β (95%CI)	Direct effect β (95%CI)	Indirect effect β (95%CI)
**Acc** – m*g* (quartile)			
BMI	−0.13 (−0.19; −0.07)	−0.09 (−0.15; −0.03)	−0.04 (−0.06; −0.02)
WC	−0.13 (−0.19; −0.07)	−0.09 (−0.15; −0.03)	−0.04 (−0.06; −0.02)
SBP	−0.13 (−0.19; −0.07)	−0.10 (−0.17; −0.04)	−0.03 (−0.05; −0.01)
DBP	−0.13 (−0.19; −0.07)	−0.07 (−0.13; −0.01)	−0.06 (−0.08; −0.03)
**MVPA** – minutes (quartile)			
BMI	−0.09 (−0.15; −0.03)	−0.08 (−0.14; −0.02)	−0.01 (−0.03; 0.01)
WC	−0.09 (−0.15; −0.03)	−0.05 (−0.11; 0.02)	−0.04 (−0.07; −0.02)
SBP	−0.09 (−0.15; −0.03)	−0.07 (−0.13; −0.01)	−0.02 (−0.04; 0.00)
DBP	−0.09 (−0.15; −0.03)	−0.06 (−0.12; 0.00)	−0.03 (−0.06; −0.01)
**Sedentary time** – minutes (quartile)			
BMI	0.11 (0.05; 0.17)	0.10 (0.04; 0.16)	0.01 (−0.01; 0.03)
WC	0.11 (0.05; 0.17)	0.10 (0.04; 0.16)	0.02 (0.00; 0.03)
SBP	0.11 (0.05; 0.17)	0.10 (0.04; 0.16)	0.01 (−0.01; 0.03)
DBP	0.11 (0.05; 0.17)	0.08 (0.02; 0.14)	0.03 (0.01; 0.06)

Acc – Physical activity evaluated by raw data accelerometer; MVPA – Moderate-to-Vigorous Physical Activity; BMI – Body Mass Index; WC – waist circumference; SBP – Systolic Blood Pressure; DBP – Diastolic Blood Pressure.

Variables included in the analysis as base confounders: sex, skin color, family income at birth, National Economic Indicator score and smoking.

Variables included in the analysis as post confounder: daily energy intake.
